# MSP-1p42-specific antibodies affect growth and development of intra-erythrocytic parasites of *Plasmodium falciparum*

**DOI:** 10.1186/1475-2875-8-183

**Published:** 2009-08-03

**Authors:** Elke S Bergmann-Leitner, Elizabeth H Duncan, Evelina Angov

**Affiliations:** 1 US Military Malaria Vaccine Program, Walter Reed Army Institute of Research, Silver Spring, MD 20910, USA

## Abstract

**Background:**

Antibodies are the main effector molecules in the defense against blood stages of the malaria parasite *Plasmodium falciparum*. Understanding the mechanisms by which vaccine-induced anti-blood stage antibodies work in protecting against malaria is essential for vaccine design and testing.

**Methods:**

The effects of MSP-1p42-specific antibodies on the development of blood stage parasites were studied using microscopy, flow cytometry and the pLDH assay. To determine allele-specific effects, if present, allele-specific antibodies and the various parasite clones representative of these alleles of MSP-1 were employed.

**Results:**

The mode of action of anti-MSP-1p42 antibodies differs among the parasite clones tested: anti-MSP-1p42 sera act mainly through invasion-inhibitory mechanisms against FVO parasites, by either preventing schizonts from rupturing or agglutinating merozoites upon their release. The same antibodies do not prevent the rupture of 3D7 schizonts; instead they agglutinate merozoites and arrest the development of young parasites at the early trophozoite stage, thus acting through both invasion- and growth inhibitory mechanisms. The second key finding is that antibodies have access to the intra-erythrocytic parasite, as evidenced by the labeling of developing merozoites with fluorochrome-conjugated anti-MSP-1p42 antibodies. Access to the parasite through this route likely allows antibodies to exert their inhibitory activities on the maturing schizonts leading to their inability to rupture and be released as infectious merozoites.

**Conclusion:**

The identification of various modes of action by which anti-MSP-1 antibodies function against the parasite during erythrocytic development emphasizes the importance of functional assays for evaluating malaria vaccines and may also open new avenues for immunotherapy and vaccine development.

## Background

Natural immunity against malaria is based on the presence of antibodies directed against the blood stage parasite, as demonstrated by passive transfer experiments of immunoglobulins [1-3]. The mode of action of blood stage-specific antibodies depends on their antigen-specificity: they can bind to merozoites, opsonize and target them towards phagocytic cells of the host [4], or prevent invasion of new erythrocytes [5]. Once infected, antibodies against the asexual blood stage antigen Pf332 inhibit the intra-erythrocytic development of *Plasmodium falciparum *[6-8]. Furthermore, antibodies against free parasitic glycosylphosphatidylinositol (GPI) can control the severity of disease by neutralizing toxic components released from ruptured infected erythrocytes [9,10].

The major merozoite surface protein -1 (MSP-1) was identified in immune complexes from merozoite lysates, which provided the rationale for developing vaccines against this antigen [11]. MSP-1 undergoes two successive proteolytic cleavage events [12]. The second processing event occurs immediately before invasion, resulting in the cleavage of the p42 molecule into a p33 and a p19 fragment. The p19 fragment remains attached to the merozoite surface through a GPI anchor [13] and is comprised of two epidermal growth factor (EGF)-like domains [14], which may have a role in the invading complex. Serological studies have provided significant evidence to suggest that immune responses directed against the C-terminus of MSP-1 (MSP-1_19 _and MSP-1_42_) are associated with immunity in preclinical models [15-18], as well as from individuals residing in endemic areas [16,19].

In the course of characterizing immune responses induced by MSP-1 vaccines, it was recognized that: (1) proteins produced by various expression systems differ in their immunogenicity and ability to induce anti-parasite activities [20,21]; (2) not all MSP-1-based vaccines induce protective immunity [22] and; (3) the degree of inhibition was dependent on the method chosen to measure invasion- and growth inhibition mediated by anti-MSP-1p42 and that only the methods that are based on cell viability and/or metabolic activity are able to accurately measure growth inhibition, while all the methods tested were equally able to detect invasion inhibition [23]. Thus, selecting the appropriate method permits differentiation between the various mechanisms by which antibodies affect parasite development.

The current study was designed to investigate the effect of anti-MSP-1p42 antibodies on intra-erythrocytic parasite development and their consequences on parasite viability and infectivity. Depending on the parasite clone, anti-MSP-1p42 antibodies prevented schizont rupturing by stalling or arresting intra-erythrocytic parasite development likely through direct interactions with intra-erythrocytic parasites within the parasitophorous vacuole. The nature of the inhibitory effect was strongly affected not only by the parasite clone, but also by the fine-specificity of the antibodies. Only antibodies that bound the p19 subunit, but not the EGF-like domain 1 or 2 subunits, displayed growth inhibitory activities indicating that protective epitopes are dependent on the tertiary structure of the molecule.

Several important lessons can be gained from these findings: (1) various allele-specific effector mechanisms can develop to the same blood stage antigen; (2) while *in vitro *assays provide a useful tool to determine vaccine efficacy, such assays need to take into account the predominant antibody-mediated effector mechanisms against a particular clone; (3) inhibitory anti-MSP-1 specific antibodies map to epitopes formed through the "properly" folded p19 subunit and not to its sub-domains [24,25]; (4) with the exclusion of potential effector cells, only macromolecules of a defined size have access to the intra-erythrocytic parasite either through the putative "parasitophorous duct" or through the "leaky" erythrocytic membrane [6,26], and thus MSP-1-specific antibodies could be used as carriers and targeting moieties for anti-malarial drugs.

## Methods

### Parasite cultures

Complete media was prepared with RPMI 1640 (Invitrogen, Carlsbad, CA) containing 25 mM HEPES, 7.5% w/v NaHCO_3 _and 10% human pooled serum (bloodtype O+). *Plasmodium falciparum *clones 3D7, FVO and CAMP were maintained and synchronized by the temperature cycling method [27]. Unless stated otherwise, cultures in the presence or absence of immune serum were set up at approximately six hours before rupture occurred (starting parasitaemia 0.3%, 1% haematocrit uninfected erythrocytes) in 96-well plates under static conditions.

#### Access of antibodies to the intra-erythrocytic parasite

To visualize the uptake of macromolecules, non-manipulated 3D7 and FVO cultures (2–3% parasitaemia at 4% haematocrit) were incubated at the late trophozoite/early schizont stage with either 2 μl of 1:100 diluted yellow-green fluorescent FluoSpheres beads (40 nm size, Molecular Probes, Eugene, OR), or diluent (BlockAid, Molecular Probes) or 1:50 diluted Alexa 488- (Molecular Probes) or PE-conjugated antisera for two hours. Samples were enriched for parasites by passing them through a column (MS+) (Miltenyi Biotech, Auburn, CA) inserted into a MiniMACS magnet (Miltenyi Biotech) at the end of the incubation time to avoid any stress related artifacts due to out-gassing.

### Antigens

*Escherichia coli*-expressed recombinant MSP-1p42(3D7) (FMP1, falciparum malaria protein 1), MSP-1p42(FVO) (FMP003) and the codon harmonized MSP1p42(FVO) (FMP010) were produced under GMP conditions as described elsewhere [15,28,29].

### Antibodies

New Zealand White rabbits (n = 8/immunization group, Spring Valley Laboratories, Poolesville, MD) were immunized subcutaneously four times with either recombinant MSP-1p42 of the FVO clone (four immunizations with 36 μg) [15] or the 3D7 clone (prime with 225 μg and three boosts with 50 μg each) [28] emulsified in complete/incomplete Freund's adjuvant (CFA/IFA). Additional experimental groups were: rabbits (n = 4) immunized subcutaneously three times with 50 μg of codon harmonized MSP-1p42(FVO) [29] in CFA/IFA and Montanide ISA-720 (Seppic, Paris, France) and rabbits (n = 4) immunized subcutaneously three times with 50 μg of reduced/alkylated MSP-1p42 (3D7) and (FVO) in CFA/IFA (control sera) [23]. Serum pools were aliquoted and stored frozen until analysis. Where indicated, immunoglobulins were purified from sera obtained after immunization with either reduced/alkylated MSP-1p42 (3D7 or FVO allele) emulsified in CFA/IFA. Immunoglobulins were purified using HiTrap Protein G columns (GE Healthcare, Piscataway, NJ) according to manufacturer's instructions and conjugated with fluorochromes following the manufacturer's instructions. Fluorochromes used were Alexa 488 or phycoerythrin (both purchased from Molecular Probes). Purified immunoglobulins were tested at concentrations normalized to the titers of the respective antisera. Both, antisera and purified immunoglobulins were tested in growth inhibition assays using recombinant MSP-1p42 antigen add-back (reversal of inhibition assay) to assure that the activity seen was antigen-specific.

### ELISA

ELISA assays were performed as previously described in detail [30].

### Affinity purification of MSP-1 subunit specific antibodies

GSH resin was coated with 2.5 mg/ml protein (GST-MSP-1p19 and GST-p19 EGF-domain 1 or 2 of either the 3D7 or the FVO allele [15]). Pooled sera from five or eight rabbits, respectively, immunized with either MSP-1p42(3D7)/FA or MSP-1p42(FVO)/FA were incubated with protein-coated GSH resin (GE Healthcare) for 1 hr at RT. Resin was washed and the bound antibodies were eluted by washing the resin three times with 75 mM glycine (pH = 3.0) and followed by two times with 0.5 M NaCl. Eluted antibodies were analysed by SDS gel electrophoresis using 4–20% SDS-PAGE gels (Invitrogen, Carlsbad, CA).

### Microscopic analysis

Cultures were harvested at various time points as indicated and blood smears were made from each well. Blood smears were fixed in methanol and stained in a 10% Giemsa solution (Sigma, St. Louis, MO) for 10 min. Slides were washed in water and allowed to air dry before analysis. Evaluation was performed at 1,000× magnification (oil-immersion) using a Nikon E400 Eclipse microscope and photographs were taken with an Olympus BX-50 microscope with digital camera (Magnafire Software). Three slides per group were evaluated by counting 2000 erythrocytes (RBC) or 100 parasitized erythrocytes (pRBCs)/slide in a blinded fashion.

### Confocal microscopy

Confocal images were taken on a Nikon E800 microscope and the laser confocal system, BioRad Radiance 2100. Thirty layers at a spacing of 0.5 μm were scanned for each image and then z-stacked (performing 3D Blind Deconvolution on the stack (AutoDeblur deconvolution software, AutoQuant Imaging Inc, Watervliet, NY)). Images were analysed at 3% laser power.

### Evaluation of invasion-/growth inhibition

Cultures for growth inhibition assays were set up at the schizont stage and cultured for one cycle as described [23]. For pLDH detection, cells were harvested, washed and frozen at -30°C until analysis. pLDH was detected and measured as described previously [31,32]. For flow cytometric analysis of growth- and invasion inhibition, 50 μl culture aliquots were stained with hydroethidine (HE) (Polysciences, Warrington, PA) or Syto-16 (Molecular Probes, Eugene, OR). Stock solutions of HE were prepared at 10 mg/ml dissolved in DMSO (Sigma) and stored at -30°C. Samples were stained by adding 500 μl of freshly diluted HE (diluted 1:200 in warm (37°C) phosphate buffered saline (PBS) (Biowhittaker, Walkersville, MD)) to the parasite suspensions and incubating for 20 min at 37°C. Syto-16 was diluted to 200 nM with PBS; a 500 ml aliquot was added to the parasite suspensions and incubated for 30 min at 37°C. Staining was stopped by transferring the samples to ice (which allowed stabilization of the staining for up to two hours) and diluted with 1 ml PBS prior to analysis. The data were acquired on a FACSCalibur flow cytometer (Becton Dickinson, San Jose, CA) using CellQuest software for acquisition and analysis.

## Results

### Immune rabbit sera raised against various MSP-1p42 proteins are growth/invasion inhibitory against homologous and heterologous clones of *P. falciparum*

**Figure 1:**
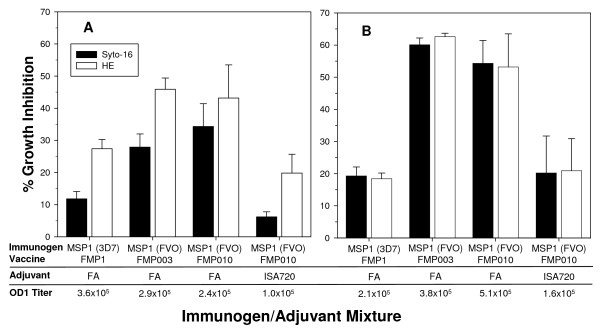
**Anti-MSP-1p42 specific sera act to inhibit either merozoite invasion or parasite growth, depending on the parasite clone**. Responses of 3D7 (Panel A) and FVO (Panel B) parasites to various anti-MSP-1p42 antisera (at 20% v/v) were tested and evaluated by determining inhibitory activity of the sera based on total parasitaemia (Syto-16; black bars) and viable parasitaemia (HE, white bars). Test sera used: MSP-1p42 (FVO)(FMP003, FMP010) or MSP-1p42 (3D7) (FMP1) in Freund's adjuvant (FA) or FMP003 and FMP010 in Montanide ISA 720. Data are the mean and SEM of three independent experiments. The ELISA data are expressed as OD 1 titers against the MSP-1p42(3D7) (Panel A) or MSP-1p42 (FVO) (Panel B) plate antigen.

While some methods used to detect *in vitro *anti-parasitic activities are able to measure invasion inhibition, only the methods that require metabolically active cells such as staining parasitized RBCs with hydroethidine (HE) or detection of pLDH in cultures are able to measure growth inhibition [23]. To determine whether immunizations with MSP-1p42 always induce growth inhibitory antibodies regardless of the adjuvant and the immunogen, rabbits were immunized with various immunogen/adjuvant combinations (Figure 1). The immunogens compared were MSP-1p42 of the 3D7 allele [28] or FVO allele (either the wild-type nucleotide sequence containing a single codon substitution (mut7) (FMP003) [15] or the full gene "codon harmonized" nucleotide sequence, FMP010 [29] adjuvanted in either Freund's adjuvant (FA) or Montanide ISA-720. Growth inhibition was evaluated by flow cytometric analyses using Syto-16 DNA dye binding for total parasitaemia (regardless of viability of pRBC) and by hydroethidine (HE) DNA dye binding (for viable parasitaemia). Anti-MSP-1p42 specific antibodies inhibited invasion (Syto-16) and growth (HE) on 3D7 parasites (Figure 1A), while the various antisera tested mainly inhibited invasion of FVO parasites (Figure 1B). Although quantitative differences in the growth inhibitory activity of the various sera were noted, no qualitative differences between the immunogen/adjuvant combinations tested were observed. As no qualitative differences were seen between FMP003 or FMP010 vaccines (both of which are based on MSP-1p42 of the FVO clone), only sera raised using the FMP003 vaccine were used in subsequent experiments. Interestingly, both FVO-based vaccines induced antibodies with higher inhibitory activities against 3D7 parasites than the homologous FMP1 vaccine. This is not necessarily reflected in the reactivity of the antibodies in the ELISA-based analysis (Figure 1). The higher GIA activities or specificities against the heterologous strain may be due to improved protein structures of the recombinant FVO proteins thus more closely resembling the native structure.

### MSP-1p42 specific antibodies cause morphological changes to parasite in *in vitro *cultures

**Figure 2:**
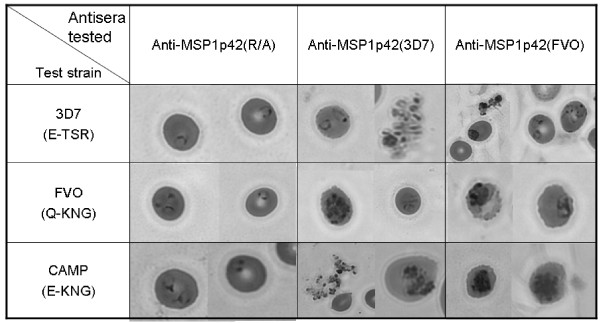
**Anti-MSP-1p42 antisera prevent the rupture of heterologous and homologous clones, FVO and CAMP/FUP, but not 3D7**. Cultures were set up with schizont stage pRBCs in the presence of sera (at 20% v/v) from rabbits immunized with either MSP-1p42(3D7) (FMP1) or MSP-1p42(FVO) (FMP003) emulsified in FA. Sera from rabbits immunized with reduced/alkylated MSP-1p42(R/A)/FA were used as the negative control. Triplicate blood smears were prepared from each culture 6 hrs after invasion was completed. Micrographs of representative parasites were taken at 1000× magnification.

3D7, FVO and CAMP/FUP schizont stage parasites were incubated with either control sera (MSP-1p42 reduced/alkylated (R/A)) or MSP-1p42 of the 3D7 or FVO allele for 10–22 hrs (i.e., 6 and 12 hrs post invasion). Representative examples of the predominant morphologies are shown in Figure 2, with quantitative evaluation in Table 1. 3D7 cultures incubated with anti-MSP-1p42 antisera contained predominantly ring stage parasites and agglutinated merozoites at six hours post-invasion (pI). Conversely, for the FVO and CAMP/FUP cultures at six hours pI, newly invaded rings and agglutinated merozoites were observed (Figure 2). Furthermore, stalled schizonts were identifiable as mature schizonts with pronounced nuclei which persist in culture several hours beyond the time point when schizonts in control cultures have ruptured and released merozoites. Stalled schizonts did not rupture throughout the experiment and were only observed in the presence of immune sera. Similar results were obtained when using either purified immunoglobulins from immunized rabbits or the serum from a malaria-exposed individual, indicating that the observed effects were not artifacts caused by either the control or immune rabbit serum.

**Table 1:** Quantitative analysis of Giemsa-stained blood smears of cultures incubated with various antisera.

(A) 6 hrs post invasion				
**Test clone**	**Antisera tested^a^**	**Proportion of phenotype [%]^b^**		
		**rings**	**schizonts**	**agg mz**
**3D7**	Control (R/A)	100	0	n
	Anti-MSP-1p42(3D7)	100	0	y
	Anti-MSP-1p42(FVO)	100	0	y
**FVO**	Control (R/A)	98.8	1.2	n
	Anti-MSP-1p42(3D7)	70.0	30.0	y
	Anti-MSP-1p42(FVO)	65.4	34.6	y
**CAMP/FUP**	Control (R/A)	97.1	2.9	n
	Anti-MSP-1p42(3D7)	79.2	20.8	y
	Anti-MSP-1p42(FVO)	74.3	25.7	y
(B) 12 hrs post invasion				
**Test clone**	**Antisera tested^a^**	**Proportion of phenotype [%]^b^**		
		**rings**	**schizonts**	**agg mz**
**3D7**	Control (R/A)	100	0	n
	Anti-MSP-1p42(3D7)	100	0	y
	Anti-MSP-1p42(FVO)	100	0	y
**FVO**	Control (R/A)	99.4	0.6	n
	Anti-MSP-1p42(3D7)	73.4	26.6	y
	Anti-MSP-1p42(FVO)	61.9	38.1	y
**CAMP/FUP**	Control (R/A)	96.6	3.4	n
	Anti-MSP-1p42(3D7)	75.2	24.8	y
	Anti-MSP-1p42(FVO)	70.1	29.9	y

^**a **^Antisera were tested at 20% (v/v) serum concentration. The control group R/A consists of pooled sera from rabbits immunized with reduced/alkylated MSP-1p42 in FA (see Materials and Methods).

^**b **^Analysis was done by counting sufficient fields to accumulate either 2,000 RBCs or 100 pRBC. The pRBC were classified and counted based on their phenotype. No attempts were made to count agglutinated merozoites (agg mz) and thus only their presence in the culture was noted as y, yes and n, no. Data shown in the table correspond to the data shown in Figure 2.

**Figure 3:**
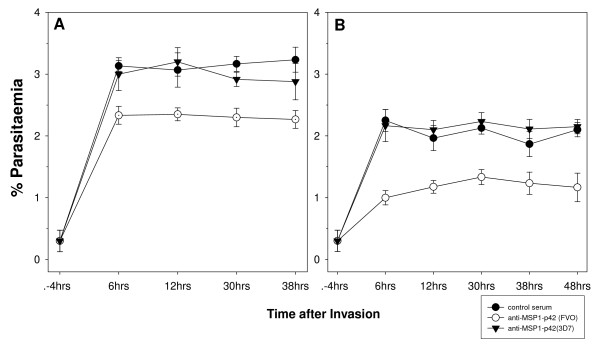
**Anti-sera raised to MSP-1p42 treated parasite cultures results in retardation of parasite development**. 3D7 (panel A) and FVO (panel B) schizonts were cultured in the presence of control serum or immune rabbit serum (at 20% v/v) raised against either reduced/alkylated MSP-1p42 (R/A, control serum) or MSP-1p42(3D7) (FMP1) or MSP-1p42(FVO) (FMP003) emulsified in FA. Parallel cultures were harvested at indicated time points after completed rupture and slides prepared in triplicates per treatment group and time point. Micrographs of representative parasites in Giemsa-stained blood smears were taken at 1000× magnification.

To test whether stalled schizonts had lost the ability to rupture, the observation time was expanded from six hours pI to the next invasion cycle (i.e., 40 hrs for 3D7, 46 hrs for FVO) (Figure 3). For these experiments, total parasitaemia was determined by evaluating Giemsa-stained blood smears from parallel cultures. As previously noted [23], this readout method only detects invasion inhibition and not growth inhibition in a single-cycle experiment. This analysis demonstrated that the phenotype of the "stalled schizont" was permanent as no spike in parasitaemia was observed in the next cycle. These stalled schizonts started to either condense or disintegrate and were not observed after more than 12 hrs pI.

### Macromolecules and antibodies have access to the intra-erythrocytic parasite

The observation that antibodies can mediate growth retardation including the stalling of schizonts indicates that antibodies can access intra-erythrocytic parasite. Previous findings suggested that the membranes of infected-erythrocytes become "leaky" prior to rupture [11], providing a route through which antibodies could enter the infected RBC. An alternative explanation is that the site of invasion on the erythrocyte membrane never completely closes, leaving a pore and a small duct extending into the parasitophorous vacuole [26]. This parasitophorous duct is used for transporting macromolecules into the parasitophorous vacuole bypassing the erythrocyte membrane [26,33].

**Figure 4:**
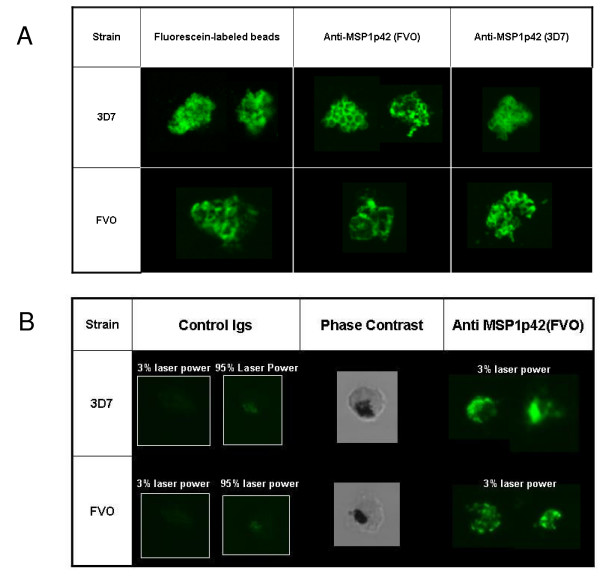
**Fluorochrome labeled beads and anti-MSP-1p42 antibodies enter viable pRBC and gain access to the intra-erythrocytic parasites**. (A) 3D7 or FVO parasite cultures (2% hct, 2–3% parasitaemia) were incubated with either Fluorescein-labeled beads or 10% Alexa 488-conjugated anti-MSP-1p42 immunoglobulins (Igs). Igs were purified from rabbit sera after immunization with MSP-1p42(FVO) (FMP003) emulsified in FA. Igs were tested at the same titer as serum assay. (B) same as for panel A, except that pRBCs were incubated with either Alexa 488-labeled Igs derived from rabbits either immunized with reduced/alkylated MSP-1p42 (negative control) or MSP-1p42(FVO) (FMP003) emulsified in FA. The confocal microscopy images were recorded at either 3% laser power (same power as the images in Panel A) or at 95% laser power (highest laser sensitivity). The center column shows the bright field image of the left column.

To address the question of whether antibodies have access to intra-erythrocytic parasites, parasite cultures were incubated with either fluorochrome-labeled beads or fluorochrome-labeled anti-MSP-1p42 antibodies for two hours and then were evaluated by confocal microscopy (Figure 4). Fluorescein-labeled beads as well as the purified labeled antibodies were detected within infected erythrocytes and in the case of the fluorescein-labeled beads, outlined a labyrinth-like structure around the intra-erythrocytic parasites similar to the structures described by Pouvelle *et al*. [33] (Figure 4A, left column). In the case of the Alexa 488-labeled MSP-1p42 specific antibodies, a grape-like staining pattern consistent with an MSP-1-like staining [34] was observed in the pRBC (Figure 4A, middle and right column). No differences between the two test clones, 3D7 and FVO, could be found with regards to either staining intensity or staining pattern. Although antibodies from both control- and immune sera were present in the pRBCs, the staining pattern with the control antibodies was only discernable at the highest setting for sensitivity (95% laser power), were highly diffuse and did not outline the parasites' nuclei similar to the immune serum (Figure 4B). The micrographs for the immune antibodies were taken at 3% laser power (at which the control antibodies did not show any staining).

### Anti-MSP-1p42 specific antibodies retard the development of intra-erythrocytic parasites

**Figure 5:**
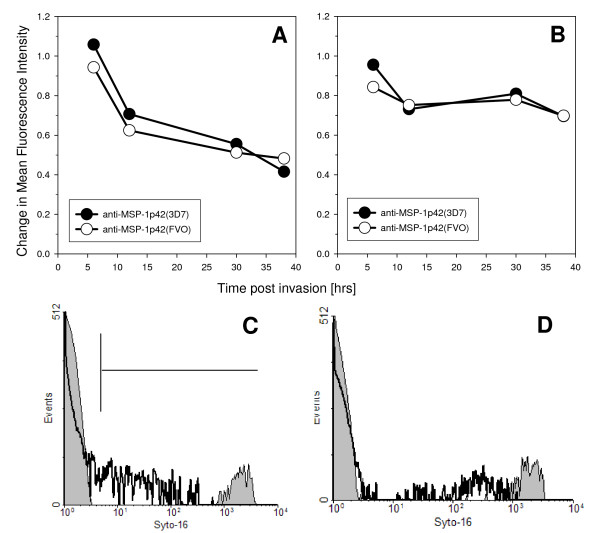
**Flow cytometric analysis reveals reduction in parasitaemia for both 3D7 and FVO parasite cultures when MSP-1p42 specific antibodies are present**. Panel A, B: Changes in DNA content of RBC infected with either 3D7 parasites (Panel A) or FVO parasites (Panel B) cultured either in the presence of anti-MSP-1p42(3D7) (FMP1) (closed circle) or anti-MSP-1p42(FVO) (FMP003) (open circle) (serum concentration = 20% v/v). Infected RBC were stained with Syto-16 and their DNA content determined by flow cytometry and compared to pRBC cultured in the presence of control sera (DNA content = 1). Panel C, D: representative histograms of 3D7 (Panel C) or FVO (Panel D) schizonts after culture with either control sera (black bold line, white area) or MSP-1p42(FVO) (FMP003) (thin line, grey area) specific antisera. Histogram analysis was used to derive the data shown in Panel A, B. Adjuvant used for all three groups was CFA/IFA. One of three representative experiments is shown.

Evaluating blood smears from cultures treated with MSP-1p42 specific antibodies taken at various time points throughout the cycle revealed that growth retardation became apparent mainly at the trophozoite stage for 3D7 parasites. The observed growth retardation was quantified by staining for parasite DNA in RBCs cultured in the presence of either control sera or immune sera. Shown in Figure 5, 3D7 (Panel A) and FVO (Panel B) parasite cultures taken at various times throughout the cycle were stained with Syto-16. The MFI of control cultures was set as 1 and any changes in MFI of the cultures treated with immune sera was calculated (Figure 5A, B). Thus, for anti-MSP-1p42-treated parasite cultures having lower DNA content than control cultures the MFI would be < 1. Lower DNA content for cultures treated with immune sera compared to control cultures at 12 hrs pI were observed for both 3D7 and FVO parasites (30–40% less in 3D7 parasites and approximately 25% reduction in FVO parasites). In addition, 3D7 parasites were consistently affected by the presence of the immune sera as there was a further reduction in DNA content. This was confirmed by evaluating these same cultures using HE, where a significant growth inhibition of 3D7 parasites was measured compared to FVO parasites starting at the early trophozoite stage [23]. FVO parasites however, appeared to be influenced by MSP-1p42 specific antibodies only at the early and the late stages of the cycle, with growth inhibitory effects undetectable by HE [23].

### MSP-1p42 and p19-specific antibodies are responsible for the functional activity in immune sera

**Figure 6:**
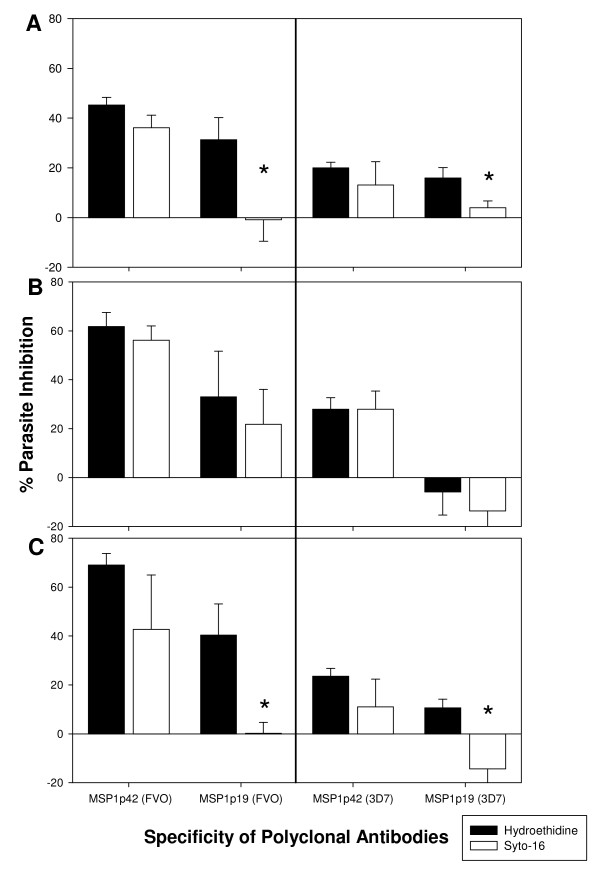
**Antibodies recognizing epitopes contained within the p19 of MSP-1p42 are responsible for inhibitory activity in immune serum**. Antibodies from sera raised against MSP-1p42 of the 3D7 (FMP1) or FVO (FMP003) allele were purified using recombinant GST-MSP-1p19 either to 3D7 or the FVO allele and then tested for their functional activity. Cultures were set up at schizont stage in the presence of indicated antibody fractions (tested at the same titers as for the respective serum assays) and then incubated for 40 hrs. Invasion inhibition was measured by Syto-16 staining; growth/invasion inhibition was measured by HE-staining and subsequent flow cytometry against 3D7 (Panel A), FVO (Panel B) and CAMP/FUP (Panel C) parasites. Data are expressed as mean percentage of inhibition from three separate experiments (SEM). Asterisk indicates statistical difference of p < 0.01 (Student's T-test) between Syto-16 and HE.

MSP-1 specific antibodies were found to be associated with reduced parasite density and clinical disease and are mainly directed to the C-terminal-MSP-1p19 portion of MSP-1 in seroepidemiological studies [16,35]. Determination of antibody fine specificities either by using an MSP-1p42 fragment specific ELISA from an *Aotus *monkey study [15] or by sub-domain affinity purifications from protective hyperimmune immunoglobulin [16] showed that the antibodies directed to EGF-like domain 2 were associated with parasitological readouts or growth inhibitory activities, respectively, while antibodies directed to the EGF-like domain 1 were associated primarily with "blocking" activities, i.e., antibodies that interfere with the activity of inhibitory antibodies [25]. To date, antibodies directed primarily to either EGF-like domain 1 or domain 2 appear to have a less clear role. Thus, experiments were designed to address whether inhibitory antibody activities induced by vaccinating rabbits with MSP-1p42 mapped to the whole MSP-1p42 molecule or to the subunits (MSP-1p19, EGF-like domain 1 or EGF-like domain 2). Affinity-purified antibodies from sera of rabbits immunized with either MSP-1p42(3D7) or MSP-1p42(FVO) were generated using various subunit fragments: MSP-1p42, MSP-1p19 and MSP-1p19 EGF domain 1 and domain 2 representative of both the 3D7 or FVO allele [15,28]. The affinity-purified subunit-specific antibodies were tested (after normalizing to their starting concentration within the serum) for their anti-parasite activity using flow cytometry (Figure 6). The inhibitory effects of the respective antibodies were evaluated by measuring total parasitaemia (Syto-16 staining, indicative of invasion inhibition) and viable parasitaemia (HE-staining, indicative of growth inhibition). These studies identified that: (1) MSP-1p42 specific antibodies act through growth- and invasion inhibitory activities. (2) Purified antibodies to MSP-1p19 act in an allele-specific manner. For example, invasion, but not growth of FVO parasites was inhibited, while growth, but not invasion of 3D7 and CAMP/FUP was inhibited by anti-MSP-1p19 antibodies. The highest level of growth inhibition observed with these antibodies was against 3D7 followed by CAMP parasites. And conversely, MSP-1p19 (FVO) purified antibodies act against FVO parasites by both invasion and growth inhibition, but do not inhibit either 3D7 or CAMP parasites by invasion inhibition (Figure 6, Left Panels A and C). Finally, (3) antibodies specific for either EGF-like domain 1 or EGF-like domain 2 for either allele had no anti-parasite activities (data not shown).

## Discussion

There is significant data in the literature supporting the development of MSP-1p42 as a blood-stage vaccine candidate primarily as antibodies directed against this antigen have been shown to protect against homologous challenge in *Aotus *monkey studies, albeit using strong adjuvants like complete Freund's/incomplete Freund's [15], as well as shown to correlate with reduced parasite burden and clinical disease from human sero-epidemiological studies (reviewed in [36]). Thus, it is crucial to understand the potential mechanisms underlying the anti-parasitic activities for this target antigen in order to design MSP-1-based vaccines with maximal vaccine efficacy. In two recent studies which compare various MSP-1-based vaccine candidates with regards to their immunogenicity and ability to induce antibodies that mediate anti-parasitic activity [20,21], the differences observed in antibody binding and functional activities induced were attributed to either the immunogens ability to induce more "biologically" relevant activities against the parasite or from the effects on the immunogens conformation as a result of the expression or purification systems utilized. This is not surprising as the quality of the immune response induced may be influenced by the quality of the protein produced. To avoid issues of expression and purification process differences, the recombinant MSP-1p42 molecules used as immunogens in the current study were all expressed similarly from *E. coli *and purified as soluble proteins [15,28,29]. Where directly comparable, no apparent differences were observed in the anti-parasitic activities induced by either the single codon mutated MSP-1p42 (FMP003) and full gene codon harmonized MSP-1p42 (FMP010) in FA or Montanide ISA 720 as adjuvant. Therefore, for at least the two adjuvants tested, the mode of action of the functional activities induced in rabbits was not adjuvant-dependent.

The present study demonstrates that antibodies directed against MSP-1p42 play a complex role during invasion and intra-erythrocytic maturation (growth) of parasites. Using comparative studies of *P. falciparum *FVO, CAMP/FUP and 3D7 parasite cultures, we made several observations: first, that the quality of the response of MSP-1p42 specific antibodies is independent of the immunogen and adjuvant used (Figure 1); second, that the "type" of response against the parasite, whether invasion or growth inhibitory, was dependent on the assay clone, for instance, invasion- and growth inhibition was observed for 3D7 and CAMP/FUP parasites while only invasion inhibition was observed for FVO parasites (Figures 1, 6); third, that only antibodies raised against MSP-1p42 or affinity-purified using either the 3D7 or FVO p19 fragments had any inhibitory activity, while antibodies affinity-purified using either the EGF-like domain 1 or 2 did not (Figure 6); fourth, that the MSP-1p19 specific antibodies component exhibited homologous allele-specific inhibitory activities that were not observed for antibodies to the total MSP-1p42; and finally, that antibodies were able to enter viable infected erythrocytes and bind to intra-erythrocytic parasites, and that likely through this action, inhibit either further development or prevent rupture of maturing schizonts (Figures 2, 5, 6).

The present study revealed various reaction patterns of blood stage parasites in response to MSP-1p42 specific antibodies: invasion inhibition mediated by preventing schizont maturation ("stalled schizonts"), schizont rupturing and agglutination of merozoites prior to or upon their release (Figure 2). The observation that anti-MSP-1p42 specific antibodies cannot prevent the rupture of 3D7 schizonts (Figure 2) is not due to a general inability of the schizonts to be stalled as anti-AMA-1 specific antibodies induced this phenotype as well (Bergmann-Leitner, unpublished observations). Preventing schizonts from rupturing permanently affected the schizonts, as no spike in parasitaemia at any later time points could be detected suggesting that the observed effect was likely not due to a transient phenotype. Growth inhibition or retardation becomes evident by progressive losses in the ability to stain DNA with HE and reductions in the levels of pLDH measured in the cultures in the presence of immune serum. Moreover, growth retardation was observed when pRBC cultures treated with either control or immune serum were compared for their DNA content as a measurement of parasite maturation (Figure 5). As suggested by the HE-staining, 3D7 parasites were more affected by the phenomenon of growth retardation than were FVO parasites. However, measuring the DNA content as a marker for maturation does not necessarily indicate that the effect of immune antibodies is persistent, i.e., even though the maturation is slowed these cultures retained similar multiplication rates as control cultures. Thus currently, the only feasible measures of growth inhibition are those methods that are based on parasite viability such as HE-staining of DNA and measurement of pLDH [23].

In an effort to better understand how MSP-1p42 specific antibodies affect intra-erythrocytic parasite development, such that schizonts can no longer rupture, it was first corroborated that antibodies, regardless of their specificity, can enter infected RBCs (Figure 4), as shown previously for other macromolecules, such as beads having diameters up to 80 nm [26,33], iron chelators [37], antisense oligonucleotides [38], plasmids [39] and antibodies [6]. In the case where these antibodies are unable to prevent invasion of erythrocytes, antibodies that are bound to parasites may be dragged into the newly invaded RBC and remain bound to the ring stage parasite during the early phases of development immediately after invasion (unpublished observation, and [40]). A recent report demonstrates that the p19 fragment of MSP-1 may be involved in the formation of the food vacuole [41]. It needs to be determined how antibody binding to p19 during and after invasion affects the role of the molecule during this crucial developmental step. In the later development it is possible that the antibody-bound MSP-1 on intra-erythrocytic parasites interferes with the ability to cause the phenotypic changes on FVO and CAMP/FUP schizonts that lead to rupture. Finally, this study addressed the question of anti-MSP-1p42 specific antibodies fine specificities and their role in mediating the various phenotypes of invasion and growth inhibition that were detected. To this end, antibodies were affinity-purified using either recombinant MSP-1p19 or the EGF-like domain 1 or 2 (representative of the 3D7 or FVO allele) contained within the p19 fragment (Figure 6). Flow cytometric analysis of parasite cultures treated with the various antibody preparations derived from rabbit immune serum revealed that only antibodies raised against the MSP-1p42 or affinity purified antibodies specific for MSP-1p19 had any inhibitory activity. These results are consistent with the findings obtained from naturally-exposed Western Kenyan adults' immune sera where using transgenic *P. falciparum *expressing MSP-1-p19 orthologs, the majority of the inhibitory activity was directed to the MSP-1p19 portion of the molecule [19,35]. Affinity purified anti-MSP-1 antibodies using EGF-like domain 1 and 2 proteins did not capture antibodies that had any inhibitory function indicating that the crucial epitopes are formed by both domains or that the epitope is larger than just one of the domains alone. This finding is in agreement with epitope mapping studies for the growth inhibitory mAb, namely 12.10 [42], where both domains were required for mAb binding [25,43]. Interestingly, MSP-1p19-purified antibodies appeared to be more allele-specific with regard to invasion inhibition than the total MSP-1p42 antibody population as MSP-1p19 (3D7) antibodies were only able to inhibit 3D7 parasites and to a lesser extent CAMP/FUP parasites and not FVO parasites. Similarly, MSP-1p19 (FVO)-purified antibodies only inhibited invasion of FVO parasites, while the effect on 3D7 and CAMP/FUP parasites was solely growth-inhibitory. Thus the mechanism of invasion inhibition may be more allele-specific than the mechanism of growth inhibition.

## Conclusion

The findings from pre-clinical studies in rabbits provide new insights into the possible mode of action of anti-MSP-1p42 specific antibodies and their broader effect on intra-erythrocytic stage parasite development and thus solicit similar characterizations of antibodies directed against other blood stage antigens. Likewise, affinity purification of MSP-1p42 specific antibodies isolated from "protected" individuals residing in endemic regions may further support and define the relevance of these findings and lead to a better understanding of the role and modes of action of these antibody activities.

## Conflict of interests

The authors declare that they have no competing interests.

## Authors' contributions

ESB-L drafted the manuscript, co-designed the study, conducted most of the experiments and performed growth inhibition assays. EHD assisted in growth inhibition assays, kinetic experiments and performed the affinity purifications. EA designed the study, directed the work and edited the manuscript. All authors have read and approved the final manuscript.

## Disclaimer

Research was conducted in compliance with the Animal Welfare Act and other federal statutes and regulations relating to animals and experiments involving animals and adheres to principles stated in the *Guide for the Care and Use of Laboratory Animals*, NRC Publication, 1996 edition.

The authors' views are private and are not to be construed as official policy of the Department of Defense or the U.S. Army.
